# Comparative analysis of cardiorespiratory fitness, bio-motor abilities, and body composition indicators among sprint kayakers of different age groups and expertise levels

**DOI:** 10.3389/fphys.2023.1259152

**Published:** 2023-09-01

**Authors:** Xiaodong Wang, Liqiu Zhao

**Affiliations:** ^1^ School of Physical Education, Shaoguan University, Shaoguan, Guangdong, China; ^2^ Department of Quality Education, Jiangsu Vocational College of Electronics and Information, Huaian, Jiangsu, China

**Keywords:** performance prediction, conditioning, aerobic fitness, muscular power, body composition, sprint kayak

## Abstract

This study compared cardiorespiratory fitness, bio-motor abilities, and body composition indexes of sprint kayakers categorized into three different age groups and two expertise levels of international- and club-level athletes. Seventy-three male juniors (*n* = 14, age = 16.2 ± 0.8), under 23 [U23 (*n* = 15, age = 20.2 ± 1.6)], professionals (*n* = 16, age = 27.1 ± 4.8), club-level (*n* = 15, age = 26.9 ± 6.6), and international-level (*n* = 13, age = 27.3 ± 3.2) sprint kayakers were studied. Cardiorespiratory fitness (assessed using incremental exercise test), 500 and 1,000-m paddling performance (assessed using kayak ergometer), upper-body power (assessed using 30 s *all-out* Wingate test) and strength (assessed through one repetition tests for bench press, cable row, and prone bench pull exercises), as well as body composition indexes (measured using dual-energy X-ray absorptiometry) were evaluated on four occasions separated by 48 h recovery. U23 and, especially, professionals indicated significantly (*p* < 0.05) greater outcomes for the majority of the cardiorespiratory fitness parameters [maximal oxygen uptake (VO_2max_), velocity corresponding to VO_2max_, Oxygen pulse, maximal ventilation, and second ventilatory threshold] and 500 and 1,000-m performance. U23 and professional sprint kayakers significantly (*p* < 0.05) differed in the first ventilatory threshold and 500, and 1,000-m performance but not in VO_2max_ or the second ventilatory threshold. Professionals also showed a lower fat mass, higher muscle mass, and higher strength (bench press, prone bench pull, and seated cable row) and power than U23 and junior kayakers. Strength and power indicators had significantly greater values in U23 athletes compared to juniors. International-level athletes also showed superior VO_2max_, velocity corresponding to VO_2max_, middle (500-m), and long-distance (1,000-m) time trial performance, strength and power, lower fat, and higher muscle mass than club-level sprint kayakers. Cardiorespiratory fitness (particularly ventilatory threshold), body composition, and muscle strength/power are the best differentiating factors for sprint kayakers of different ages and expertise levels. These findings could aid coaches in prescribing training programs focusing on improving determining factors in paddling performance, as well as in predicting performance and identifying talent.

## 1 Introduction

Achieving the highest level of performance in any sport is a gradual and comprehensive endeavor that extends over multiple years (Alejo et al., 2022), requiring continuous improvement of sport-specific characteristics from a junior level, progressing to under 23 (U23), and ultimately getting the professional level. Physiological attributes, anthropometric variables, bio-motor abilities, and body composition parameters affecting sprint kayaking performance have already been well elucidated (Fry and Morton, 1991; Byrnes and Kearney, 1997; Bishop, 2000; van Someren and Palmer, 2003; van Someren and Howatson, 2008; Michael et al., 2009; Buglione et al., 2011; McKean and Burkett, 2014; Borges et al., 2015; Hamano et al., 2015; López-Plaza et al., 2017; Paquette et al., 2018; Pickett et al., 2018; Coleho et al., 2020; Kukic et al., 2022; Gäbler et al., 2023). However, the information regarding the difference between age categories and expertise levels is limited. Understanding the actual physiological and performance disparities among athletes of various age groups and expertise levels could help to identify particular attributes that need to be developed from younger ages to professional levels to enhance the likelihood of achieving high-level performance (Sheykhlouvand et al., 2015; Foster et al., 2022).

Sprint kayak is an Olympic event on a flat-water course with official races in World championships set over four distances of 200-m (∼38 s), 500-m (∼100 s), 1,000-m (∼220 s), and 5,000-m (∼1,290 s) for men senior category (International Canoe Federation)^1^. Races are completed individually and in crews of up to four. Kayakers compete in a seated position, propelling themselves forward using a double-blade paddle. Research indicates that sprint kayak performance mainly depends on upper-body anaerobic and aerobic power (Borges et al., 2015; López-Plaza et al., 2017; Barzegar et al., 2021; Sheykhlouvand et al., 2022). For instance, “using the accumulated oxygen deficit method, the contribution of aerobic metabolism to different distances in highly-trained kayakers has been estimated at 37%, 64%–78%, and 85%–87% for 200-m, 500-m, and 1,000-m, respectively (Byrnes and Kearney, 1997; Zamparo et al., 1999; Zouhla et al., 2012)”. Middle-distance events (500-m and 1,000-m) kayak sprint performance is strongly correlated to maximum oxygen uptake (VO_2max_) and lactate threshold (Zamparo et al., 1999; Paquette et al., 2018). By contrast, short-distance 200-m performance is not related to VO_2max_ and lactate threshold but upper-body anaerobic power/capacity, distinguishing international-level kayakers from national-level athletes (van Someren and Palmer, 2003).

Enhanced upper-body strength and muscular endurance also significantly determine sprint performance (Forbes and Sheykhlouvand, 2016; Sheykhlouvand et al., 2022). Enhancing the pulling motion during paddling strokes leads to a steady augmentation of the force during the entire pulling phase, resulting in better speed maintenance (Ualí et al., 2012; McKean and Burkett, 2014). Kayakers must generate considerable average power during each stroke and apply significant forces on the blade of the paddle while propelling forward to achieve the highest average boat velocity (Michael et al., 2009; Kukić et al., 2022). Muscle mass significantly affects force outcomes per paddling stroke (Kukić et al., 2022). Also, higher body fatness increases the drag force and reduces the paddling efficacy (Michael et al., 2009; McDonnell et al., 2012). Hence, when planning long-, medium- and short-term training, it is crucial to consider the body composition of sprint kayakers (Borges et al., 2015).

Although previous studies have identified various contributing attributes to sprint kayak performance, reviewing the literature indicates that no previous study has directly compared these parameters among sprint kayakers of varying age groups and expertise levels. Accordingly, this study aimed to compare cardiorespiratory fitness, bio-motor abilities, and body composition indicators among sprint kayakers of different age groups (junior, U23, professional) and expertise levels (international-level vs. club-level). We hypothesized the presence of a linear progression from junior to professional levels for the specified variables. Additionally, individuals competing at the international level will exhibit superior physiological and performance capabilities compared to sprint kayakers participating at the club level.

## 2 Materials and methods

### 2.1 Participants

Seventy-three male sprint kayakers classified as junior (*n* = 14, age = 16.2 ± 0.8), U23 (*n* = 15, age = 20.2 ± 1.6), professionals (*n* = 16, age = 27.1 ± 4.8), club-level (*n* = 15, age = 26.9 ± 6.6), and international-level (*n* = 13, age = 27.3 ± 3.2) gave their written informed consent and volunteered to participate. All participants actively engaged in national and international competitions, including Asian or World Championships, representing their country of origin as members of their national teams. Kayakers underwent the assessment approximately 1 month after the last event of the pre-season phase. All participants were medication-free and with no musculoskeletal injuries or other conditions hindering their participation. The procedures followed during the study adhered to the ethical guidelines outlined in the Helsinki Declaration and were approved by the ethical committee of Shaoguan University, China.

### 2.2 Study design

The research was conducted using a cross-sectional observational design. Kayakers attended the lab on four different days, separated by 48 h recovery between testing days to assess body composition and cardiorespiratory fitness (first session), muscular strength (second session), upper-body anaerobic power (third session), and paddling performance using a kayak ergometer (Dansprint, Hvidovre, Denmark). 500, and 1,000-m paddling time trials were completed on the last assessment day, and the tests were separated with 2 h of recovery. All tests were conducted under the same condition. Participants were instructed to follow their habitual dietary pattern and abstain from caffeine, alcohol, and severe exercise in the 24 h period preceding the testing sessions (Gharaat et al., 2020).

### 2.3 Body composition assessment

Participants’ body composition [bone mineral content (BMC), body mass, and muscle and fat mass] was measured using a dual-energy X-ray absorptiometry (DXA, Lunar Prodigy Advance; GE-Medical, Systems, Madison, WI, United States). Participants’ stature was determined by a wall-mounted digital stadiometer (Charder, HM200D, Taichung, Taiwan).

### 2.4 Incremental exercise test using gas collection system

Participants completed a graded exercise test on a kayak ergometer (Dansprint PRO, Hvidovre, Denmark). Following a standardized warm-up consisting of a 3-min paddling at ∼85% HR_max_ followed by two 15 s accelerations interspersed with 45 s rest and 2 standing starts of 24 strokes with 45 s rest between, ending with a 3-min paddling at 85% HR_max_ (Borges et al., 2015), the trial commenced at 6 km·h^−1^ and was followed by 1 km·h^−1^ increments every 1 min until volitional exhaustion (Sheykhlouvand et al., 2022; Sheykhlouvand et al., 2018a; Sheykhlouvand et al., 2018b). Expired air was continuously recorded during the test using a breath-by-breath gas collection system (MetaLyzer 3B-R2, Cortex, Germany). VO_2max_ was determined as the highest 30 s value in the trial if: 1) the VO_2_ approached a plateau; 2) RER of >1.1; 3) reaching ≥ 90% age-predicted HR; 4) visible fatigue (Sheykhlouvand et al., 2016b; Fereshtian et al., 2017; Sheykhlouvand and Forbes, 2017). Two independent experts localized the first and second ventilatory thresholds (VT_1_ and VT_2_). VT_1_ was established as the point where an elevation in the V_E_/VO_2_ and end-tidal O_2_ tension (P_ET_O_2_) occurred with no simultaneous elevation in V_E_/VCO_2_. VT_2_ identification criterion was the continuous elevation in the V_E_/VO_2_ and V_E_/VCO_2_ ratio curves concerning the decrease in P_ET_O_2_ (Alejo et al., 2022).

### 2.5 Muscular strength

Maximal dynamic strength in the bench press (BP), seated cable row (CR), and prone bench pull (PBP) movements were determined by the evaluation of one repetition maximum (1RM). The test began with a warm-up comprising a 5-min paddling on a kayak ergometer at a self-selected low-intensity pace, a 5-min joint mobilization exercise for the upper body, and a standardized weight lifting warm-up (Earle, 2006). Then the kayakers completed 3–5 1RM sets with a 4-min of recovery between sets to determine 1RM. The heaviest load lifted by the participant with proper exercise technique was considered to represent his 1RM (Earle, 2006).

### 2.6 Upper-body anaerobic power

Participants completed a 30-s *all-out* upper-body Wingate test on a mechanically braked arm ergometer (891E; Monark, Vansbro, Sweden) to determine upper-body peak power output (PPO) and average power output (APO). Kayakers were instructed to exert maximum effort by rapidly cranking against the internal resistance of the ergometer. Within 3 s, a load corresponding to 0.075 kg per kilogram of their body mass (Forbes et al., 2014) was immediately applied. The participants received verbal motivation to crank at their maximum speed during the entire Wingate test (Sheykhlouvand et al., 2016a). The device software was utilized to calculate the PPO and the APO.

### 2.7 Paddling performance

Using the same kayak ergometer (Dansprint), participants completed time trials in 500, and 1,000-m distances with 2 h of recovery between tests. Following a standardized warm-up consisting of a 3-min paddling at ∼85% HR_max_ followed by two 15 s accelerations interspersed with 45 s rest and 2 standing starts of 24 strokes with 45 s rest between, ending with a 3-min paddling at 85% HR_max_ (Borges et al., 2015), participants were instructed to paddle distances with maximal effort, and the ergometer recorded the times.

### 2.8 Statistical analysis

With an effect size of 0.8, assuming an alpha error of 0.05, β of 0.08, and using G*Power software (Faul et al., 2007), the sample size was estimated to be at least seven participants in each group. However, the sample size was later increased in groups, with the possibility of some participants dropping out during data collection and to enhance the power of the test. SPSS software version 25.0 (Statistical Package for Social Science) analyzed the data. Descriptive statistics were reported by Mean ± SD values. The normality of the distribution (Kolmogorov–Smirnov test) and homogeneity (Levene’s test) of the data were checked in advance for statistical analysis. A one-way analysis of variance (ANOVA) with Bonferroni posthoc analyzed the difference between categories (age groups and expertise levels). Effect size was calculated using Cohen’s d (*d*). The α level for significance was set at 0.05 and type I error was controlled by adding 95% confidence intervals (CI).

## 3 Results

Tables 1–6 represent the difference between age groups and expertise levels for aerobic fitness, 500- and 1,000-m paddling performance, strength and power, and body composition indicators.

**TABLE 1:** Difference in cardiorespiratory fitness indicators and time trial performance between age groups.

Variable	Groups			Main *p* value	Effect size		
	Junior (*n* = 14)	*p* value	Pro (*n* = 16)		Junior vs. U23	Junior vs. Pro	U23 vs. Pro
VO_2max_ (mL/kg/min)	44.6 ± 2.6	52.3 ± 3.6	54.7 ± 2.6	<0.001***	2.452***	3.884***	0.764
vVO_2max_ (km/h)	14.8 ± 1.0	16.1 ± 0.9	16.3 ± 0.1	<0.001***	1.366**	1.500**	0.210
HR at VO_2max_ (%)	84.8 ± 3.0	84.2 ± 4.3	85.8 ± 3.0	0.986	0.161	0.333	0.431
VO_2_/HR (mL/b/min)	20.0 ± 1.5	23.7 ± 2.0	24.9 ± 2.0	<0.001***	2.093***	2.771***	0.600
V_E_ (L/min)	151.6 ± 9.3	181.7 ± 25.2	189.7 ± 19.6	<0.001***	1.584**	2.483***	0.354
VT_1_ (%VO_2max_)	69.0 ± 4.6	70.8 ± 2.6	75.1 ± 4.6	0.002**	0.481	1.326**	1.151**
VT_2_ (%VO_2max_)	88.0 ± 4.3	90.3 ± 3.4	90.7 ± 4.1	0.967	0.593	0.642	0.106
500-m TT (sec)	125.9 ± 3.9	122.9 ± 3.6	115.7 ± 2.3	<0.001***	0.799	2.185***	2.383***
1,000-m TT (sec)	247.2 ± 4.5	244.0 ± 6.7	230.9 ± 4.6	<0.001***	0.561	2.582***	2.279***

VO_2max_, maximum oxygen uptake; vVO_2max_, velocity associated with VO_2max_; HR, heart rate; V_E_, maximal ventilation; VT_1_, first ventilatory threshold; VT_2_, second ventilatory threshold; TT, time trial. *p < 0.05; **p < 0.01; ***p < 0.001.

**TABLE 2:** Difference in cardiorespiratory fitness indicators and time trial performance between different expertise levels.

Variable	Groups		Main *p* value	Effect size
	Club-level (*n* = 15)	*p* value		Club-level vs. International-level
VO_2max_ (mL/kg/min)	49.1 ± 3.9	56.7 ± 1.7	<0.001***	2.526***
vVO_2max_ (km/h)	15.7 ± 1.0	17.1 ± 0.9	0.007**	1.471**
HR at VO_2max_ (%)	83.4 ± 3.6	84.6 ± 3.6	0.986	0.724
VO_2_/HR (mL/b/min)	23.4 ± 2.4	23.4 ± 2.8	0.572	0.000
V_E_ (L/min)	173.5 ± 25.5	197.3 ± 18.7	0.034*	1.064*
VT_1_ (%VO_2max_)	71.7 ± 4.8	74.4 ± 3.9	0.827	0.617
VT_2_ (%VO_2max_)	89.6 ± 3.8	92.6 ± 2.7	0.518	0.936
500-m TT (sec)	123.3 ± 4.0	112.8 ± 1.0	<0.001***	2.601***
1,000-m TT (sec)	246.8 ± 3.2	225.1 ± 4.5	<0.001***	2.557***

VO_2max_, maximum oxygen uptake; vVO_2max_, velocity associated with VO_2max_; HR, heart rate; V_E_, maximal ventilation; VT_1_, first ventilatory threshold; VT_2_, second ventilatory threshold; TT, time trial. *p < 0.05; **p < 0.01; ***p < 0.001.

**TABLE 3:** Difference in indicators of bio-motor abilities between age groups.

Variable	Groups			Main *p* value	Effect size		
	Junior (*n* = 14)	*p* value	Pro (*n* = 16)		Junior vs. U23	Junior vs. Pro	U23 vs. Pro
PPO (W)	381.6 ± 62.9	473.8 ± 46.9	589.6 ± 56.2	<0.001***	1.662***	2.487***	2.237***
PPO (W/kg)	4.7 ± 0.8	5.8 ± 0.6	7.0 ± 0.7	<0.001***	1.555***	2.059***	1.841***
APO (W)	302.3 ± 50.8	375.3 ± 36.8	428.6 ± 40.2	<0.001***	1.645***	2.757***	1.383**
APO (W/kg)	3.7 ± 0.6	4.6 ± 0.5	5.1 ± 0.5	<0.001***	1.629***	2.535***	0.911
BP 1RM (kg)	92.7 ± 6.5	103.0 ± 7.2	129.0 ± 7.2	<0.001***	1.501*	2.292***	1.611***
BP 1RM [kg/body mass (kg)]	1.14 ± 0.08	1.26 ± 0.09	1.54 ± 0.08	<0.001***	1.409*	2.112***	2.936***
PBP 1RM (kg)	84.3 ± 5.8	96.7 ± 7.7	117.5 ± 7.1	<0.001***	1.819***	2.121***	2.808***
PBP 1RM [kg/body mass (kg)]	1.04 ± 0.07	1.20 ± 0.11	1.40 ± 0.08	<0.001***	1.735***	2.789***	2.079***
CR 1RM (kg)	92.1 ± 6.4	104.7 ± 7.7	123.7 ± 6.7	<0.001***	1.779***	2.823***	2.632***
CR 1RM [kg/body mass (kg)]	1.14 ± 0.07	1.28 ± 0.11	1.48 ± 0.09	<0.001***	1.676**	2.217***	1.990***

PPO, peak power output; APO, average power output; BP, bench press; PBP, prone bench pull; CR, cable row; 1RM, one repetition maximum; W, watts. *p < 0.05; **p < 0.01; ***p < 0.001.

**TABLE 4:** Difference in indicators of bio-motor abilities between different expertise levels.

Variable	Groups		Main *p* value	Effect size
	Club-level (*n* = 15)	*p* value		Club-level vs. international-level
PPO (W)	500.7 ± 52.6	612.0 ± 38.2	<0.001***	2.421***
PPO (W/kg)	6.1 ± 0.7	7.3 ± 0.4	<0.001***	2.105***
APO (W)	388.5 ± 30.9	434.3 ± 38.9	0.035*	1.303*
APO (W/kg)	4.7 ± 0.4	5.2 ± 0.5	0.143	0.943
BP 1RM (kg)	109.3 ± 11.6	130.4 ± 8.2	<0.001***	2.100***
BP 1RM [kg/body mass (kg)]	1.32 ± 0.14	1.56 ± 0.09	<0.001***	2.039***
PBP 1RM (kg)	101.3 ± 9.1	118.8 ± 6.5	<0.001***	2.213***
PBP 1RM [kg/body mass (kg)]	1.23 ± 0.11	1.43 ± 0.07	<0.001***	2.169***
CR 1RM (kg)	109.7 ± 8.9	125.0 ± 6.1	<0.001***	2.005***
CR 1RM [kg/body mass (kg)]	1.33 ± 0.11	1.50 ± 0.08	<0.001***	1.767***

PPO, peak power output; APO, average power output; BP, bench press; PBP, prone bench pull; CR, cable row; 1RM, one repetition maximum; W, watts. *p < 0.05; **p < 0.01; ***p < 0.001.

**TABLE 5:** Difference in body composition indicators between age groups.

Variable	Groups			Main *p* value	Effect size		
	Junior (*n* = 14)	*p* value	Pro (*n* = 16)		Junior vs. U23	Junior vs. Pro	U23 vs. Pro
Height (cm)	180.5 ± 2.5	181.0 ± 2.4	180.5 ± 2.4	0.911	0.204	0.000	0.208
Body mass (kg)	80.9 ± 3.2	81.3 ± 2.5	83.5 ± 2.1	0.121	0.139	0.960	0.952
BMI (kg/m^2^)	24.8 ± 1.1	24.9 ± 0.9	25.6 ± 0.6	0.141	0.696	0.902	0.915
BMC (kg)	3.6 ± 0.5	3.6 ± 0.4	3.8 ± 0.6	0.654	0.000	0.362	0.392
BMC (%)	4.4 ± 0.6	4.5 ± 0.5	4.6 ± 0.7	0.893	0.181	0.306	0.164
Fat mass (kg)	9.4 ± 1.3	9.2 ± 0.9	7.0 ± 1.0	<0.001***	0.178	2.069***	2.312***
Fat mass (%)	11.6 ± 1.7	11.3 ± 1.2	8.3 ± 1.2	<0.001***	0.203	2.242***	2.500***
Muscle mass (kg)	67.8 ± 3.3	68.5 ± 2.6	72.7 ± 2.2	<0.001***	0.235	1.747***	1.743***
Muscle mass (%)	83.8 ± 1.7	84.2 ± 1.5	87.0 ± 1.5	<0.001***	0.249	1.996***	1.866***

BMI, body mass index; BMC, bone mineral content. ***p < 0.001.

**TABLE 6:** Difference in body composition indicators between different expertise levels.

Variable	Groups		Main *p* value	Effect size
	Club-level (*n* = 15)	*p* value		Club-level vs. International-level
Height (cm)	180.4 ± 1.9	181.1 ± 1.9	0.968	0.368
Body mass (kg)	82.2 ± 2.2	83.2 ± 2.1	0.929	0.464
BMI (kg/m^2^)	25.2 ± 0.9	25.3 ± 0.6	0.999	0.131
BMC (kg)	3.7 ± 0.5	3.6 ± 0.5	1.000	0.200
BMC (%)	4.5 ± 0.6	4.4 ± 0.6	1.000	0.166
Fat mass (kg)	9.3 ± 1.4	7.6 ± 1.0	0.003**	1.397**
Fat mass (%)	11.3 ± 1.6	9.2 ± 1.3	0.002**	1.440**
Muscle mass (kg)	69.1 ± 1.6	72.9 ± 2.8	0.023*	1.666*
Muscle mass (%)	84.1 ± 1.5	86.4 ± 1.5	0.003**	1.533**

BMI, body mass index; BMC, bone mineral content. ***p < 0.001.

### 3.1 Cardiorespiratory fitness and paddling performance

Results showed a significant group effect for most cardiorespiratory fitness indexes and paddling performance, with a consistent increase from junior category to professional levels in most of the analyzed parameters (Table 1). U23 kayakers indicated significantly greater VO_2max_ (*d* = 2.452, CI = 4.51–10.84), vVO_2max_ (*d* = 1.366, CI = 0.72–2.77), VO_2_/HR (*d* = 2.093, CI = 1.57–5.81), and V_E_ (*d* = 1.584, CI = 8.51–51.67) than juniors. Professional athletes showed significantly greater VO_2max_ (*d* = 3.884, CI = 6.93–13.27), vVO_2max_ (*d* = 1.500, CI = 0.52–2.54), VO_2_/HR (*d* = 2.771, CI = 2.85–7.05), V_E_ (*d* = 2.483, CI = 16.79–59.30), VT_1_ (*d* = 1.326, CI = 1.55–10.57), and better 500-m TT (*d* = 2.185, CI = −13.45 to −6.90), and 1,000-m TT (*d* = 2.583, CI = −21.27 to −11.28) performance than junior kayakers. Professional and U23 sprint kayakers significantly differed in VT_1_ (*d* = 1.151, CI = 1.16–8.69), 500-m TT (*d* = 2.383, CI = −10.41 to −3.97), and 1,000-m TT (*d* = 2.279, CI = −17.97 to −8.16) performance. No differences were found for VO_2max_ or VT_2_. %VO_2max_ corresponding to VT_1_ and VT_2_ indicated no linear increase from juniors to professionals. Also, the international-level athletes indicated significantly greater VO_2max_ (*d* = 2.526, CI = 4.36–10.92), vVO_2max_ (*d* = 1.471, CI = 0.27–1.99), and V_E_ (*d* = 1.064, CI = 1.84–45.85), and significantly better 500-m TT (*d* = 2.601, CI = −13.81 to −7.03) and 1,000-m TT (*d* = 2.557, CI = −26.96 to −16.62) performances when compared to club-level sprint kayakers (Table 2).

### 3.2 Bio-motor abilities

Results indicated a significant group effect for all strength and power variables with a consistent linear association (i.e., consistent elevation of strength/power from junior levels to professionals) for the strength and power indicators. More specifically, U23 kayakers indicated greater PPO (W) (*d* = 1.662, CI = 37.77–146.69), PPO (W/kg) (*d* = 1.555, CI = 0.41–1.79), APO (W) (*d* = 1.645, CI = 31.38–114.58), APO (W/kg) (*d* = 1.629, CI = 0.31–1.42), BP 1RM (kg) (*d* = 1.501, CI = 1.61–18.96), BP 1RM (kg/kg) (*d* = 1.409, CI = 0.13–0.23), PBP (kg) (*d* = 1.819, CI = 4.69–20.07), PBP (kg/kg) (*d* = 1.735, CI = 0.09–0.24), CR 1RM (kg) (*d* = 1.779, CI = 4.94–20.11), and CR 1RM (kg/kg) (*d* = 1.676, CI = 0.08–0.24) than juniors (Table 3).

Professional kayakers indicated greater PPO (W) vs. juniors (*d* = 2.487, CI = 154.36–261.62) and vs. U23 (*d* = 2.237, CI = 63.09–168.43), PPO (W/kg) vs. juniors (*d* = 2.059, CI = 0.66–2.05) and vs. U23 (*d* = 2.841, CI = 0.54–1.88), APO (W) vs. juniors (*d* = 2.757, CI = 85.30–165.24) and vs. U23 (*d* = 2.383, CI = 13.06–93.52), APO (W/kg) vs. juniors (*d* = 2.535, CI = 0.83–1.92), BP 1RM (kg) vs. juniors (*d* = 2.292, CI = 27.81–44.89) and vs. U23 (*d* = 1.611, CI = 17.67–34.45), BP 1RM (kg/kg) vs. juniors (*d* = 2.112, CI = 0.29–0.51) and vs. U23 (*d* = 2.936, CI = 0.17–0.38), PBP 1RM (kg) vs. juniors (*d* = 2.121, CI = 25.64–40.79) and vs. U23 (*d* = 2.808, CI = 13.41–28.27), PBP 1RM (kg/kg) vs. juniors (*d* = 2.789, CI = 0.27–0.46) and vs. U23 (*d* = 2.079, CI = 0.12–0.31), CR 1RM (kg) vs. juniors (*d* = 2.823, CI = 24.14–39.08) and vs. U23 (*d* = 2.632, CI = 11.75–26.42), and CR 1RM (kg/kg) vs. juniors (*d* = 2.217, CI = 0.24–0.43) and vs. U23 (*d* = 1.990, CI = 0.09–0.29) (Table 3).

Also, PPO (W) (*d* = 2.421, CI = 55.73–166.80), PPO (W/kg) (*d* = 2.105, CI = 0.54–1.96), APO (W) (*d* = 1.303, CI = 0.09–1.06), BP 1RM (kg) (*d* = 2.100, CI = 12.21–29.90), BP 1RM (kg/kg) (*d* = 1.039, CI = 0.12–0.24), PBP (kg) (*d* = 2.217, CI = 9.67–25.35), PBP (kg/kg) (*d* = 2.169, CI = 0.11–0.29), CR 1RM (kg) (*d* = 2.005, CI = 7.60–23.07), and CR 1RM (kg/kg) (*d* = 1.767, CI = 0.09–0.26) values were significantly higher in international-level athletes than in club-level sprint kayakers (Table 4).

### 3.3 Body composition

Results indicated no significant group effect on height, body mass, BMI, and BMC. However, we found a significant group effect for absolute and relative fat mass and muscle mass, with professional kayakers indicating a significantly lower absolute fat mas vs. juniors (*d* = 2.069, CI = −3.66 to −1.24) and vs. U23 (*d* = 2.312, CI = −3.66 to −0.99), lower relative fat mas vs. juniors (*d* = 2.242, CI = −4.76 to −1.87) and vs. U23 (*d* = 2.500, CI = −4.33 to −1.49), higher absolute muscle mass vs. juniors (*d* = 1.747, CI = 2.16–7.47) and vs. U23 (*d* = 1.743, CI = 1.60–6.81), and higher relative muscle mass vs. juniors (*d* = 1.996, CI = 1.53–4.73) and vs. U23 (*d* = 1.866, CI = 1.23–4.37) sprint kayakers (Table 5).

Also, international-level athletes indicated lower absolute fat mass (*d* = 1.397, CI = −2.94 to −0.44) and relative fat mass (*d* = 1.440, CI = −3.64 to −0.64) and greater absolute muscle mass (*d* = 1.666, CI = 0.58–5.51) and relative muscle mass (*d* = 1.533, CI = 0.58–3.89) than club-level sprint kayakers (Table 6).

## 4 Discussion

The most striking findings of this study were that U23 athletes, especially professional sprint kayakers, displayed significant variations in cardiorespiratory fitness indicators (e.g., VO_2max_, vVO_2max_, VO_2_/HR, V_E_) and paddling performance, compared to junior athletes. However, no disparities were observed in major indicators like VO_2max_ when comparing U23 athletes and professional sprint kayakers. There were evident strength and power differences between professionals and the other two categories, consisting of relative and absolute PPO, APO, and 1RM in BP, PBP, and CR. Professional athletes also displayed lower body fat and higher muscle mass than juniors and U23 athletes. Athletes competing at the international level exhibited superior aerobic power, bio-motor abilities, and paddling performance compared to their club-level counterparts. Results revealed a significant contrast in body composition indicators, with international-level athletes displaying lower body fat and higher muscle mass.

Cardiorespiratory fitness indicators have been widely used as determinants of sprint kayak performance (Borges et al., 2015; Paquette et al., 2018; Sheykhlouvand et al., 2022; Du and Tao, 2023). In addition to 500 and 1,000-m TT, most of the assessed cardiorespiratory fitness parameters had different values between junior kayakers and the other age groups. Still, only some variables, such as VT_1_ (but not VO_2max_), varied between U23 and professional sprint kayakers. Research has indicated that, by accounting for ∼79% variance in 500-m paddling time, anaerobic threshold is considered an effective factor on 500-m TT (Bishop, 2000). The significance of the lactate threshold (Winchcombe et al., 2019) and ventilatory threshold (Michael et al., 2009) in paddling performance has been emphasized by additional research, with the proportion of anaerobic energy metabolism contribution changing based on the distance. However, no linear continuity (i.e., elevation from junior to professional levels) was found for %VO_2max_ at which VT_1_ and VT_2_ occurred. A proportion of VO_2max_ that an athlete can maintain for a specified time has traditionally been considered a measure of endurance (Billat, 1996; Joyner and Coyle, 2008). Research controversially indicates that trained athletes may exhibit a ventilatory threshold (overall equivalent to lactate thresholds) at a higher percentage of VO_2max_ compared to untrained individuals (Alejo et al., 2022). In the present study, we found no difference for VT_2_ between age categories, but professionals attained a higher percentage of VO_2max_ at VT_1_ than junior and U23 kayakers. However, when assessing endurance performance, it is recommended not to rely solely on this parameter to predict endurance performance accurately. Instead, it should be used with other factors, like vVO_2max_ or peak power output (Støa et al., 2020).

In longer distances (i.e., 1,000-m) also, aerobic power (indicated by VO_2max_) plays a vital role in successful performance (Michael et al., 2009; Zouhla et al., 2012). Regardless of the distance covered (500 or 1,000-m), the anaerobic pathway is the primary energy source at the start of the race, but it decreases and becomes almost exclusively aerobic toward the end (Zouhla et al., 2012). However, the contribution of aerobic metabolism during 1,000-m races is significantly greater than that of 500-m kayaking events (Fry and Morton, 1991; Zouhla et al., 2012; Paquette et al., 2018). Beyond the magnitude of aerobic and anaerobic contribution, the capacity of muscle to extract O_2_, which is independent of VO_2max_, is considered a more vital determinant of performance (Paquette et al., 2018). It is generally accepted that VO_2max_ is determined by O_2_ delivery (i.e., central component) and O_2_ extraction by the active muscles (i.e., central component) (Sheykhlouvand et al., 2022; Paquette et al., 2018 indicated that muscle capacity to extract O_2_ strongly influences paddling performance and could be considered better predictor of performance than VO_2max_ in sprint kayakers. In this study, we have not evaluated muscle oxygenation, and we cannot speculate in this regard, but O_2_ extraction, could be considered an effective part of the peripheral component of aerobic fitness, affecting sprint kayak performance (Paquette et al., 2018).

Contrary to aerobic fitness, strength and power showed a remarkable difference between U23 and professionals, making them a more sensitive performance marker than VO_2max_. The importance of muscular strength and power production in kayaking performance has already been well-established (Michael et al., 2009; McKean and Burkett, 2014; Pickett et al., 2018). To optimize the average velocity of the boat and sprint performance, the athlete needs to generate considerable average power during each stroke and apply substantial average forces to the paddle blade while propelling forward (Messias et al., 2018; Kukic et al., 2022). Improved sprint kayak performance could be facilitated through an increase in the power-to-weight ratio of the athlete and a decrease in opposing drag forces imposed by air and water (Michael et al., 2009; McKean and Burkett, 2014). Standard and well-designed boats also can decrease both aerodynamic and hydrodynamic forces on the kayak and facilitate the movement of the kayak through the water (McKenzie and Berglund, 2019).

In line with the mentioned findings, we found a significantly different power-to-weight ratio between professionals and other age groups and U23 and junior kayakers, indicating a linear increase from the junior category to professional levels. Higher relative power could be partly attributed to greater strength and muscle mass observed in professionals compared to U23 and juniors (Michael et al., 2009). Despite a similar body mass observed between age groups, professional sprint kayakers had a greater muscle mass compared to U23 and junior athletes. The positive association between higher muscle mass and better force and power outputs of kayak stroke has already been well elucidated (Hamano et al., 2015; Kukic et al., 2022). Hence, body composition could be considered a main differentiating parameter between sprint kayakers of different age groups.

In accordance with our hypothesis, international-level sprint kayakers exhibited better VO_2max_, ventilatory threshold, muscular strength and power, body composition, and paddling performance. Provided such a vast difference, it could be recommended that to improve paddling performance, club-level athletes should focus on enhancing cardiorespiratory fitness, bio-motor abilities, and body composition to optimize their paddling performance.

Some limitations of the present study should be noted. Due to the limited presence of dedicated athletes competing across various distances, the arrangement of paddlers was not structured according to their specific racing distances. Moreover, the study’s cross-sectional nature prevents making conclusions about performance prediction, even though distinctions among groups were noted. As a result, longitudinal research is necessary to ascertain the precise utility of these variables in accurately predicting performance among young kayakers.

## 5 Conclusion

In conclusion, speed and endurance indicators of sprint kayaking (VO_2max_, VT_1_ and VT_2_, PPO, APO, strength, and muscle mass) appear to be the primary distinguishing factor between junior sprint kayakers and the higher age categories. Significant differences in cardiorespiratory fitness indicators were also observed between U23 and professional kayak sprinters, with the latter indicating better 500 and 1,000-m TT performance, muscular strength and power, VT_1_, but without differences in VO_2max_, and VT_2_. International-level athletes also showed superior cardiorespiratory fitness, bio-motor abilities, and body composition indicators than club-level sprint kayakers. While further longitudinal studies are required to validate these results, the current findings could aid coaches in prescribing training programs focusing on improving determining factors in paddling performance, as well as in predicting performance and identifying talent.

## Data Availability

The raw data supporting the conclusion of this article will be made available by the authors, without undue reservation.

## Footnotes

1
www.canoeicf.com

## References

[B1] Alejo L. B. Montalvo-Pérez A. Valenzuela P. L. Revuelta C. Ozcoidi L. M. de la Calle V. (2022). Comparative analysis of endurance, strength and body composition indicators in professional, under-23 and junior cyclists. Front. Physiol. 13, 945552. 10.3389/fphys.2022.945552 35991188 PMC9388719

[B2] Barzegar H. Arazi H. Mohsebbi H. Sheykhlouvand M. Forbes S. C. (2021). Caffeine co-ingested with carbohydrate on performance recovery in national level paddlers: A randomized, double-blind, crossover, placebo-controlled trial. J. Sport. Med. Phys. Fit. 62, 337–342. 10.23736/S0022-4707.21.12125-5 34498818

[B3] Billat L. V. Use of blood lactate measurements for prediction of exercise performance and for control of training. Recommendations for long-distance running. (1996). Recommendations for long-distance running. Sports. Med. 22(3), 157–175. 10.2165/00007256-199622030-00003 8883213

[B4] Bishop D. (2000). Physiological predictors of flat-water kayak performance in women. Eur. J. Appl. Physiol. 82 (1-2), 91–97. 10.1007/s004210050656 10879448

[B5] Borges T. O. Dascombe B. Bullock N. Coutts A. J. (2015). Physiological characteristics of well-trained junior sprint kayak athletes. Int. J. Sports. Physiol. Perform. 10, 593–599. 10.1123/ijspp.2014-0292 25473923

[B6] Buglione A. Lazzer S. Colli R. Introini E. Di Prampero P. E. (2011). Energetics of best performances in elite kayakers and canoeists. Med. Sci. Sports. Exerc. 43 (5), 877–884. 10.1249/MSS.0b013e3181fdfdb7 20962692

[B7] Byrnes W. C. Kearney J. T. (1997). Aerobic and anaerobic contributions during simulated canoe/kayak sprint events. Med. Sci. Sports. Exerc. 29 (5), S220. 10.1097/00005768-199705001-01254

[B8] Coelho B. C. Nakamura F. Y. Morgado M. C. Alves F. Di Baldassarre A. Flatt A. (2020). Prediction of simulated 1,000 m kayak ergometer performance in young athletes. Front. Public. Health 8, 526477. 10.3389/fpubh.2020.526477 33553080 PMC7855298

[B9] Du G. Tao T. (2023). Effects of a paddling-based high-intensity interval training prescribed using anaerobic speed reserve on sprint kayak performance. Front. Physiol. 13, 1077172. 10.3389/fphys.2022.1077172 36685190 PMC9848400

[B10] Earle R. W. (2006). “Weight training exercise prescription,” in Essentials of personal training symposium workbook (Lincoln, NE: NSCA Certification Commission), 3–39.

[B11] Faul F. Erdfelder E. Lang A. G. Buchner A. (2007). G∗Power 3: A flexible statistical power analysis program for the social, behavioral, and biomedical sciences. *Behav. Res*. Methods 39, 175–191. 10.3758/bf03193146 17695343

[B12] Fereshtian S. Sheykhlouvand M. Forbes S. Agha-Alinejad H. Gharaat M. (2017). Physiological and performance responses to high-intensity interval training in female inline speed skaters. Apunts. Med. L’esport. 52, 131–138. 10.1016/j.apunts.2017.06.003

[B13] Forbes S. C. Kennedy M. D. Boule N. B. Bell G. (2014). Determination of the optimal load setting for arm crank anaerobic testing in men and women. Int. J. Sports. Med. 35, 835–839. 10.1055/s-0034-1368789 24920563

[B14] Forbes S. C. Sheykhlouvand M. (2016). A review of the physiological demands and nutritional strategies for canoe polo athletes. Sports. Nutr. Ther. 1, 116. 10.4172/2473-6449.1000116

[B15] Foster C. Barroso R. Beneke R. Bok D. Boullosa D. Casado A. (2022). How to succeed as an athlete: what we know, what we need to know. Int. J. Sports Physiol. Perform. 17, 333–334. 10.1123/ijspp.2021-0541 35168195

[B16] Fry R. W. Morton A. R. (1991). Physiological and kinanthropometric attributes of elite flatwater kayakists. Med. Sci. Sports. Exerc. 23 (11), 1297–1301. 10.1249/00005768-199111000-00016 1766347

[B17] Gäbler M. Prieske O. Elferink-Gemser M. T. Hortobágyi T. Warnke T. Granacher U. (2023). Measures of physical fitness improve prediction of kayak and canoe sprint performance in young kayakers and canoeists. J. Strength. Cond. Res. 37 (6), 1264–1270. 10.1519/JSC.0000000000004055 34027911

[B18] Gharaat M. A. Sheykhlouvand M. Eidi L. A. (2020). Performance and recovery: effects of caffeine on a 2000-m rowing ergometer. Sport. Sci. Health. 16, 531–542. 10.1007/s11332-020-00643-5

[B19] Hamano S. Ochi E. Tsuchiya Y. Muramatsu E. Suzukawa K. Igawa S. (2015). Relationship between performance test and body composition/physical strength characteristic in sprint canoe and kayak paddlers. J. Sports. Med. 6, 191–199. 10.2147/OAJSM.S82295 PMC4480586 26150737

[B20] Joyner M. J. Coyle E. F. (2008). Endurance exercise performance: the physiology of champions. J. Physiol. 586, 35–44. 10.1113/jphysiol.2007.143834 17901124 PMC2375555

[B21] Kukić F. Petrović M. Greco G. Cataldi S. Fischetti F. (2022). Association of anthropometrics and body composition with maximal and relative force and power of kayak stroke in competitive kayak athletes. Int. J. Environ. Res. Public. Health. 19 (5), 2977. 10.3390/ijerph19052977 35270669 PMC8909934

[B22] López-Plaza D. Alacid F. Muyor J. M. López-Miñarro P. Á. (2017). Sprint kayaking and canoeing performance prediction based on the relationship between maturity status, anthropometry and physical fitness in young elite paddlers. J. Sports. Sci. 35 (11), 1083–1090. 10.1080/02640414.2016.1210817 27433884

[B23] McDonnell L. K. Hume P. A. Nolte V. (2012). An observational model for biomechanical assessment of sprint kayaking technique. Sports. Biomech. 11, 507–523. 10.1080/14763141.2012.724701 23259240

[B24] McKean M. R. Burkett B. J. (2014). The influence of upper-body strength on flat-water sprint kayak performance in elite athletes. Int. J. Sports. Physiol. Perform. 9, 707–714. 10.1123/ijspp.2013-0301 24231254

[B25] McKenzie D. Berglund B. (2019). Handbook of sports medicine and science. Canoeing: John Wiley & Sons.

[B26] Messias L. H. D. dos Reis I. G. M. Ferrari H. G. Gobatto C. A. Serra C. C. S. Papoti M. (2018). Novel paddle stroke analysis for elite slalom kayakers: relationship with force parameters. PLoS. ONE 13, e0192835. 10.1371/journal.pone.0192835 29489872 PMC5831033

[B27] Michael J. S. Smith R. Rooney K. B. (2009). Determinants of kayak paddling performance. Sports. Biomech. 8, 167–179. 10.1080/14763140902745019 19705767

[B28] Paquette M. Bieuzen F. Billaut F. (2018). Muscle oxygenation rather than VO2max as a strong predictor of performance in sprint canoe-kayak. Int. J. Sports. Physiol. Perform. 19, 1299–1307. 10.1123/ijspp.2018-0077 29745773

[B29] Pickett C. W. Nosaka K. Zois J. Hopkins W. G. Blazevich A. J. (2018). Maximal upper-body strength and oxygen uptake are associated with performance in high-level 200-m sprint kayakers. J. Strength. Cond. Res. 32, 3186–3192. 10.1519/jsc.0000000000002398 29283928

[B30] Sheykhlouvand M. Arazi H. Astorino T. A. Suzuki K. (2022). Effects of a new form of resistance-type high-intensity interval training on cardiac structure, hemodynamics, and physiological and performance adaptations in well-trained kayak sprint athletes. Front. Physiol. 13, 850768. 10.3389/fphys.2022.850768 35360225 PMC8960736

[B31] Sheykhlouvand M. Forbes S. C. (2017). Aerobic capacities, anaerobic power, and anthropometric characteristics of elite female canoe polo players based on playing position. Sport. Sci. Health. 14, 19–24. 10.1007/s11332-017-0395-0

[B32] Sheykhlouvand M. Gharaat M. Bishop P. Khalili E. Karami E. Fereshtian S. (2015). Anthropometric, physiological, and performance characteristics of elite canoe polo players. Psychol. Neurosci. 8, 257–266. 10.1037/pne0000013

[B33] Sheykhlouvand M. Gharaat M. Khalili E. Agha-Alinejad H. (2016b). The effect of high-intensity interval training on ventilatory threshold and aerobic power in well-trained canoe polo athletes. Sci. Sports. 31, 283–289. 10.1016/j.scispo.2016.02.007

[B34] Sheykhlouvand M. Gharaat, 5 Khalili M. Agha-Alinejad E. Rahmaninia H. Arazi F. (2018a). Low-Volume high-intensity interval versus continuous endurance training: effects on hematological and cardiorespiratory system adaptations in professional canoe polo athletes. J. Strength. Cond. Res. 32, 1852–1860. 10.1519/JSC.0000000000002112 28700514

[B35] Sheykhlouvand M. Khalili E. Agha-Alinejad H. Gharaat M. A. (2016a). Hormonal and physiological adaptations to high-intensity interval training in professional male canoe polo athletes. J. Strength. Cond. Res. 30, 859–866. 10.1519/JSC.0000000000001161 26349044

[B36] Sheykhlouvand M. Khalili E. Gharaat M. Arazi H. Khalafi M. Tarverdizadeh B. (2018b). Practical model of low-volume paddling-based sprint interval training improves aerobic and anaerobic performances in professional female canoe polo athletes. J. Strength. Cond. Res. 32, 2375–2382. 10.1519/JSC.0000000000002152 29239986

[B43] Støa E. M. Helgerud J. Rønnestad B. R. Hansen J. Ellefsen S. Støren Ø. (2020). Factors influencing running velocity at lactate threshold in male and female runners at different levels of performance. Front. Physiol. 11, 585267. 10.3389/fphys.2020.585267 33250778 PMC7672120

[B37] Ualí I. Herrero A. J. Garatachea N. Marín P. J. Alvear-Ordenes I. GarcíaLópez D. (2012). Maximal strength on different resistance training rowing exercises predicts start phase performance in elite kayakers. J. Strength. Cond. Res. 26, 941–946. 10.1519/JSC.0b013e31822e58f8 22446667

[B38] van Someren K. A. Howatson G. (2008). Prediction of flatwater kayaking performance. Int. J. Sports. Physiol. Perform. 3 (2), 207–218. 10.1123/ijspp.3.2.207 19208929

[B39] van Someren K. A. Palmer G. S. (2003). Prediction of 200-m sprint kayaking performance. Can. J. Appl. Physiol. 28 (4), 505–517. 10.1139/h03-039 12904631

[B40] Winchcombe C. E. Binnie M. J. Doyle M. M. Hogan C. Peeling P. (2019). Development of an on-water graded exercise test for flat-water sprint kayak athletes. Int. J. Sports. Physiol. Perform. 14, 1244–1249. 10.1123/ijspp.2018-0717 30860403

[B41] Zamparo P. Capelli C. Guerrini G. (1999). Energetics of kayaking at submaximal and maximal speeds. Eur. J. Appl. Physiol. Occup. Physiol. 80 (6), 542–548. 10.1007/s004210050632 10541920

[B42] Zouhla H. Le Douairon Lahaye S. Ben Abderrahaman A. Minter G. Herbez R. Castagna C. (2012). Energy system contribution to Olympic distances in flat water kayaking (500 and 1,000 m) in highly trained subjects. J. Strength. Cond. Res. 26 (3), 825–831. 10.1519/JSC.0b013e31822766f7 22297414

